# Tratamento Direcionado ao Sistema Renina-Angiotensina-Aldosterona na Obesidade

**DOI:** 10.36660/abc.20200345

**Published:** 2020-07-28

**Authors:** Ariane Vieira Scarlatelli Macedo

**Affiliations:** 1 Irmandade da Santa Casa de Misericórdia de São Paulo São PauloSP Brasil Irmandade da Santa Casa de Misericórdia de São Paulo – Cardiologia,São Paulo, SP – Brasil; 2 Hospital Rede Dor São Luiz Jabaquara São Paulo Brasil Hospital Rede Dor São Luiz Jabaquara - Cardiologia, São Paulo – Brasil

**Keywords:** Obesidade, Doenças Cardiovasculares, Prevalência, Insuficiência Cardíaca, Hipertrofia Ventricular Esquerda, Sistema Renina-Angiotensina

*Minieditorial referente ao artigo: Bloqueio de Receptores AT_1_ Melhora o Desempenho Funcional Miocárdico na Obesidade*

A obesidade tem aumentado em todo o mundo, levando a um crescimento acentuado da prevalência de doenças cardiovasculares (DCV).^1^ A obesidade, principalmente a classificada como grave, provoca mudanças hemodinâmicas que contribuem para alterar a morfologia cardíaca, podendo predispor ao comprometimento da função ventricular e à insuficiência cardíaca. Essas mudanças incluem um estado de alto débito cardíaco na maioria dos casos, além de hipertrofia ventricular esquerda (HVE) e disfunção diastólica do ventrículo esquerdo (VE). Estudos experimentais e algumas investigações em humanos sugerem que alterações metabólicas e neuro-hormonais específicas geralmente associadas com a obesidade podem resultar em mudanças na estrutura e função cardíaca.^2^

Adipócitos são uma rica fonte de angiotensinogênio, angiotensina I e enzima conversora de angiotensina, contribuindo para a produção de angiotensina II. A geração desses hormônios é aditiva aos componentes do sistema renina-angiotensina-aldosterona (SRAA) produzidos sistemicamente.^3^ A obesidade é caracterizada pela ativação excessiva do SRAA. Esta ativação tem muitas implicações relativas à estrutura e à função cardíaca. A mais crítica é o efeito vasoconstritor da angiotensina II, que causa o desenvolvimento da hipertensão arterial sistêmica (HAS) em pacientes obesos, além de induzir o aumento da pós-carga do VE em indivíduos normotensos obesos.^4^

A angiotensina II atua como um fator de crescimento que afeta tanto o músculo liso vascular quanto o miocárdio. Portanto, a ativação excessiva do SRAA influencia o desenvolvimento de hipertrofia miocárdica e do músculo liso vascular.^2^ Quadros relacionados à ativação do SRAA têm sido associadas com doenças metabólicas e do coração. A ativação do SRAA também parece promover resistência à insulina e hiperinsulinemia por meio de vários mecanismos. Este cenário pode levar a um aumento da atividade do sistema nervoso simpático (SNS), contribuindo ainda mais para o desenvolvimento da HVE.^5^ Inibidores da enzima conversora de angiotensina (IECA) e bloqueadores do receptor de angiotensina II (BRA) são frequentemente usados para tratar a hipertensão associada à obesidade.

Em um experimento anterior, a intervenção com BRA (Losartan) resultou na inibição dos mecanismos moleculares de resistência à insulina no miocárdio, melhorando a contratilidade cardíaca. Recentemente, o uso de bloqueadores do receptor tipo 1 da angiotensina II (AT1) causou melhora da função mitocondrial em ratos obesos resistentes à insulina em um modelo experimental.^6^

Não está claro se o tratamento direcionado ao SRAA promove uma melhora mais significativa dos desfechos cardiovasculares em pacientes normotensos obesos em relação aos magros.

Nesta edição da ABC, Oliveira Junior, et al.,^7^ apresentam os resultados de um estudo em animais que avaliou a influência de bloqueadores de AT1 na morfologia e desempenho cardíaco por meio da análise in vitro do músculo papilar de ratos com obesidade induzida por dieta hiperlipídica. Eles hipotetizaram que a obesidade pode estar associada a alterações no desempenho funcional do miocárdio, sustentada sob estímulos diferentes, e que essas respostas podem ser atenuadas pelo antagonismo de receptores AT1. Ratos Wistar foram submetidos a controle de kcal/g ou dieta hiperlipídica por 20 semanas. Quatro grupos foram avaliados: Controle (CO), Obesos (OB), Controle com Losartan (CL) e Obesos com Losartan (OL). Os grupos CL e OL receberam Losartan (30 mg/kg/dia). Posteriormente, foram analisadas a composição corporal, a pressão arterial sistólica (PAS) e as variáveis ecocardiográficas. Os autores observaram que a obesidade estava associada com alterações de PAS e hipertrofia cardíaca. Eles também identificaram mudanças no desempenho sistólico e disfunção do músculo papilar em ratos obesos. A intervenção com o antagonista do receptor AT1 reduziu a maioria desses efeitos. Os resultados deste elegante estudo experimental reforçam a importância de intervenções cardiovasculares focadas em pacientes obesos.

Uma meta-análise de 28 ensaios clínicos randomizados envolvendo 2.403 pacientes com HAS (faixa etária média de 44–67 anos) mostrou que a regressão da HVE com medicamentos anti-hipertensivos foi maior nos grupos sobrepeso e obesidade em comparação aos indivíduos com peso normal. Mesmo com menor redução da PAS, a HVE melhorou em pacientes obesos e com sobrepeso em relação ao grupo com peso normal (p<0,001) inicial em todos os grupos). O tratamento com inibidores do sistema renina-angiotensina foi o mais eficaz na redução da HVE em indivíduos obesos e com sobrepeso (p<0,001).^6^

A obesidade é uma doença crônica e complexa baseada em adiposidade, cujo controle é direcionado tanto às complicações relacionadas ao peso quanto à adiposidade para melhorar a saúde geral. A perda de peso voluntária e substancial pode reverter muitas das alterações hemodinâmicas, neuro-hormonais e metabólicas associadas à obesidade.^8^ Intervenções médicas, como o uso de BRA, juntamente com a perda de peso, podem resultar em reversão do remodelamento cardíaco e melhorar a função ventricular e a qualidade de vida de pacientes obesos.^9^
