# Supplementary material for: Tratamento Direcionado ao Sistema Renina-Angiotensina-Aldosterona na Obesidade
Source: Arq Bras Cardiol. 2020 Jul 28;115(1):29–30. [Article in Portuguese] doi: 10.36660/abc.20200345 (PMC8384313; doi:10.36660/abc.20200345)
Supplement: Translation

# Translation: Targeting the Renin-Angiotensin-Aldosterone System in Obesity

**Authors:** Ariane Vieira Scarlatelli Macedo

**Affiliations:** 1 Brazil Irmandade da Santa Casa de Misericórdia de São Paulo – Cardiologia, São Paulo, SP – Brazil; 2 Brazil Hospital Rede Dor São Luiz Jabaquara - Cardiologia, São Paulo – Brazil

**Keywords:** Obesity, Cardiovascular Diseases, Prevalence, Heart Failure, Left Ventricular Hypertrofy, Renin-Angiotensin System

*Short Editorial related to the article: AT_1_Receptor Blockade Improves Myocardial Functional Performance in Obesity*

Obesity has been increasing across the globe and markedly raising the prevalence of cardiovascular diseases (CVD).^1^ Obesity, particularly severe obesity, causes hemodynamic alterations that contribute to changing cardiac morphology, which may predispose to impaired ventricular function and heart failure. These alterations include a high cardiac output state in most cases, as well as left ventricular hypertrophy (LVH) and left ventricular (LV) diastolic dysfunction. Experimental studies and some investigations on humans propose that specific neurohormonal and metabolic alterations generally associated with obesity may lead to changes in cardiac structure and function.^2^

Adipocytes are a rich source of angiotensinogen, angiotensin I, and angiotensin-converting enzyme, contributing to the production of angiotensin II. The generation of these hormones is additive to systemically-produced components of the renin-angiotensin-aldosterone system (RAAS).^3^ Obesity is characterized by enhanced RAAS activation. Such activation has many implications concerning cardiac structure and function. The most critical is the vasoconstrictive effect of angiotensin II, which results in the development of hypertension (HTN) in obese patients, in addition to inducing an increase in LV afterload in normotensive-obese patients.^4^

Angiotensin II is a growth factor that affects both vascular smooth muscle and myocardium. Therefore, enhanced RAAS activation influences the development of vascular smooth muscle and myocardial hypertrophy.^2^ Conditions related to RAAS activation have been associated with metabolic disorders and heart diseases. RAAS activation also seems to promote insulin resistance and hyperinsulinemia by various mechanisms. This scenario may lead to increased sympathetic nervous system (SNS) activity, further contributing to the development of LVH.^5^ Angiotensin-converting enzyme inhibitors (ACEI) and angiotensin II receptor blockers (ARB) are commonly used to treat obesity hypertension.

In a previous experiment, intervention with ARB (Losartan) resulted in the inhibition of molecular mechanisms of myocardial insulin resistance, improving heart contractility. Recently, the use of angiotensin II receptor type 1 (AT1) blockade resulted in improved mitochondrial function in obese insulin-resistant rats in an experimental model.^6^

Whether targeting the RAAS in normotensive obese patients improves cardiovascular outcomes to a greater extent than in normotensive lean patients is uncertain.

In this issue of ABC, Silva-Junior and co-authors^7^ present the results of their animal study assessing the influence of AT1 blockade on cardiac morphology and performance using in vitro papillary muscle analysis in rats with high-fat diet-induced obesity. They hypothesize that obesity might be associated with changes in myocardial functional performance, sustained under different stimuli, and that these responses could be attenuated by AT1 receptor antagonism. Wistar rats were submitted to kcal/g control or high-fat diet for 20 weeks. Four groups were assessed: Control (CO), Obese (OB), Control Losartan (CL), and Obese Losartan (OL). The CL and OL groups received Losartan (30 mg/kg/day). Afterward, body composition, systolic blood pressure (SBP), and echocardiographic variables were analyzed. The authors found that obesity was associated with SBP changes and cardiac hypertrophy. They also identified changes in systolic performance and disorders of papillary muscle function in obese rats. The AT1 receptor antagonist intervention reduced most of these effects.

The results of this elegant experimental study reinforce the importance of cardiovascular interventions focused on obese patients.

A meta-analysis of 28 randomized clinical trials comprising 2,403 patients with HTN (mean age range 44–67 years) showed that LVH regression with antihypertensive drugs was greater in the overweight and obesity groups than in normal-weight subjects. Even with a smaller reduction in SBP, LVH improved in obese and overweight groups compared to the normal-weight group (p<0.001 at baseline in all groups). Renin-angiotensin system inhibitor therapy was the most effective treatment for reducing LVH in overweight and obese patients (p<0.001).^6^

Obesity is a complex adiposity-based chronic disease, whose management targets both weight-related complications and adiposity to improve overall health. Substantial voluntary weight loss can reverse many of the hemodynamic, neurohormonal, and metabolic changes associated with obesity.^8^ Medical interventions, such as ARB, along with weight loss, may result in reverse cardiac remodeling and improve the ventricular function and the quality of life in obese patients.^9^
